# Supplementary material for: The impact of serum potassium ion variability on 28-day mortality in ICU patients
Source: PLoS One. 2024 Nov 4;19(11):e0310046. doi: 10.1371/journal.pone.0310046 (PMC11534218; doi:10.1371/journal.pone.0310046)
Supplement: S1 Appendix — (PDF) [file pone.0310046.s001.pdf]

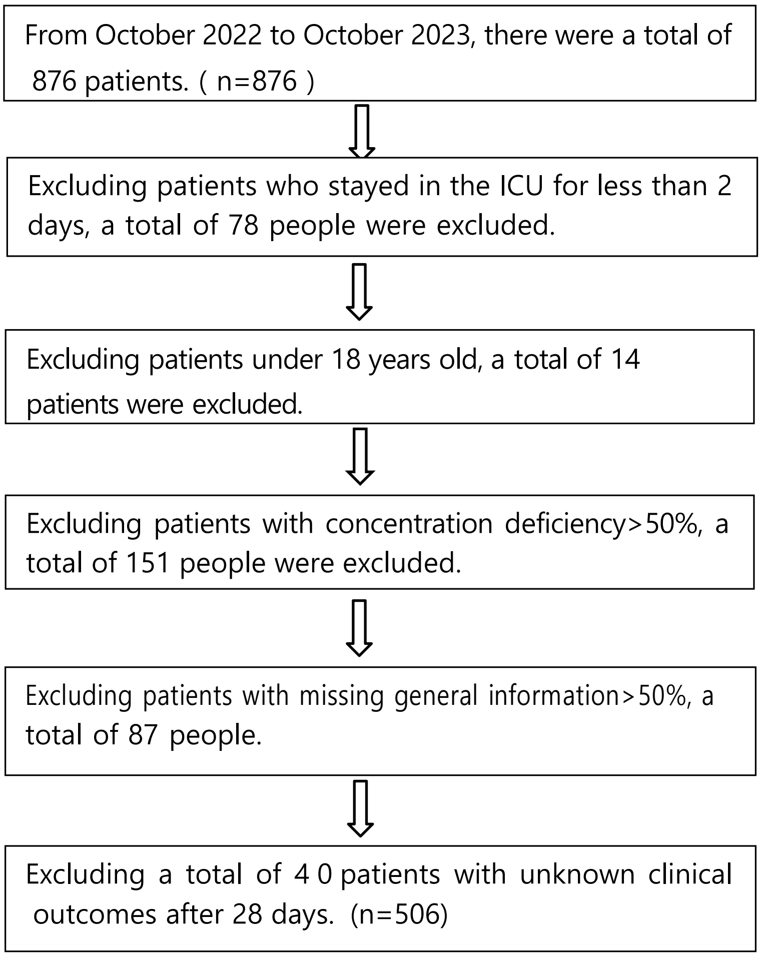
From October 2022 to October 2023, there were a total of 876 patients. (n=876)
Excluding patients who stayed in the ICU for less than 2 days, a total of 78 people were excluded.
Excluding patients under 18 years old, a total of 14 patients were excluded.
Excluding patients with concentration deficiency>50%, a total of 151 people were excluded.
Excluding patients with missing general information>50%, a total of 87 people.
Excluding a total of 4 0 patients with unknown clinical outcomes after 28 days. (n=506)
