# Supplementary material for: The impact of serum potassium ion variability on 28-day mortality in ICU patients
Source: PLoS One. 2024 Nov 4;19(11):e0310046. doi: 10.1371/journal.pone.0310046 (PMC11534218; doi:10.1371/journal.pone.0310046)
Supplement: S2 Appendix — (PDF) [file pone.0310046.s002.pdf]

Appendix 2: additional description of missing potassium measures

Table 1: Characteristics of potassium supplementation without insertion

Variable	Result
Number of patients, n	506
Number of potassium measures, n	12099
Number of missing potassium measures, n	6166
Number of patients with missing potassium, n	258
Potassium follow-up, days	28
Potassium measures per patient, n	24[4-56]

Table 2 : Characteristics of patients with and without missing potassium measures

	No Missing potassium (n=191)	Missing potassium (n=315)	p
Death, n(%)	107(57)	98(31)	0. 00
Total CV Of Potassium(%)	13(4～35)	12(2～41)	0. 02
Total SD Of Potassium	0. 53(0. 15～1. 46)	0. 49(0. 05～2. 8)	0. 07
Heart Rate, beats per minute	101(56～168)	99(46～194)	0. 28
Systolic Pressure, mmHg	125(50～192)	131(69～221)	0. 02
Diastolic Pressure, mmHg	73(36～130)	73(33～139)	0. 55
Mean Arterial Pressure, mmHg	91(41～146)	92(45～166)	0. 43
Potassium Minimum Value, mmol/L	3. 2(2. 1～4. 6)	3. 29(1. 7～4. 5)	0. 04
Potassium Maximum Value, mmol/L	5. 3(3. 9～9. 1)	5. 16(3. 2～9. 6)	0. 07
Potassium Average Value, mmol/L	4. 14(3. 32～5. 39)	4. 14(3. 15～6. 8)	0. 00
Male, n(%)	144(76)	219(70)	0. 00
Age, years	57(18～93)	59(18～101)	0. 00
ICU hospitalization days	17(2～85)	19(3～99)	0. 00
SOFA	8(2～18)	7(2～17)	0. 00
Glucose SD	1. 95(0～7. 25)	1. 86(0～13. 23)	0. 00
Glucose CV(%)	20(0～57)	20(0～73)	0. 00
Glucose Average , mmol/L	9. 7(3. 5～20)	9. 2(3～24. 2)	0. 00
Glucose Maximum , mmol/L	12. 27(0～27. 1)	11. 5(0～41. 6)	0. 00
Glucose Minimum, mmol/L	6. 88(0～13. 9)	6. 36(0～17. 1)	0. 00
Urine output	1390(0～4550)	1539(0～4950)	0. 00
Hemodialysis, n(%)	63(33)	57(18)	0. 00

Insulin, u	18(0～120)	12(0～124)	0.00
Potassium Chloride, g	0.9(0～10)	0.9(0～10)	0.45
Furosemide, mg	14(0～120)	10(0～100)	0.00
PH	7.4(6.85～7.61)	7.42(6.96～7.63)	0.00
Oxygenation Index, mmHg	228(33～649)	227(31～598)	0.00
Creatinine, μmol/L	197(16～1226)	133(17～1705)	0.00
Bilirubin, μmol/L	36(2～378)	28(2～337)	0.00
Platelet，10*9/L	180(3～546)	220(2～3206)	0.00
Coronary Heart Disease，n(%)	15(8)	38(12)	0.33
Cardiac Dysfunction，n(%)	15(8)	18(6)	0.00
Hypertension，n(%)	37（20）	87(28)	0.00
Cerebrovascular Diseases，n(%)	13（7）	17(5)	0.00
Chronic Obstructive Pulmonary Disease，n(%)	5（3）	3(1)	0.00
Diabetes，n(%)	17（20）	47(15)	0.00
Renal Insufficiency，n(%)	94（50）	124(40)	0.00
Shock，n(%)	81（43）	96(30)	0.00

Table 2 : There are differences in SOFA, ICU days, and other data between patients with potassium deficiency and those without potassium deficiency. Stratified analysis of patients with potassium deficiency suggests that the data of patients with different deficiency ratios (10%, 20%, 30%, 40%, 50%) are still consistent, and the statistical model remains significant after MI.

Table 3 : Comparison between pre MI and post MI

Missing percent	Mean of estimate	Mean of bias	Mean of RMSE
0%	4.14	-	-
10%	4.14	0.04	0.62
20%	4.14	0.04	0.65
30%	4.14	0.04	0.66
40%	4.14	0.04	0.66
50%	4.14	0.04	0.66
