# Supplementary material for: The impact of serum potassium ion variability on 28-day mortality in ICU patients
Source: PLoS One. 2024 Nov 4;19(11):e0310046. doi: 10.1371/journal.pone.0310046 (PMC11534218; doi:10.1371/journal.pone.0310046)
Supplement: S3 Appendix — (PDF) [file pone.0310046.s003.pdf]

Appendix 3 Comparison of potassium variability grouping and potassium concentration grouping

Table 1 : Comparison of potassium variability groups

	Q1 (CV≤9.17%） Average （Range）	Q2 (9.17%＜ CV≤11.43%） Average（Range）	Q3 (11.43%＜ CV≤14.37%） Average（Range）	Q4 (CV＞14.37%） Average（Range）
Death, n(%)	27(21)	51(40)	63(49)	70(54)
Total CV Of Potassium(%)	7 (2～9)	10 (9～11)	13 (11～14)	18 (14～45)
Total SD Of Potassium	0.29 (0.05～0.43)	0.43 (0.33～0.59)	0.53 (0.41～0.73)	0.79 (0.48～2.64)
Heart Rate, beats per minute	96 (46～194)	99 (50～153)	102 (56～160)	103 (57～171)
Systolic Pressure, mmHg	128 (50～201)	134 (69～208)	127 (72～221)	124 (60～190)
Diastolic Pressure, mmHg	72 (37～120)	74 (33～129)	72 (36～139)	71 (39～130)
Mean Arterial Pressure, mmHg	91(41～142)	94(45～151)	91(56～166)	89(50～144)
Potassium Minimum, mmol/L	3.48(2.6～4.6)	3.33(2.4～4.3)	3.16(1.9～4.3)	3.03(1.7～4.5)
Potassium Maximum , mmol/L	4.59(3.2～5.8)	5.01(4.1～6.8)	5.29(3.9～6.9)	5.99(4.3～9.6)
Hyperkalemia, n(%)	0(0)	7(5)	13(10)	58(45)
Hypokalemia, n(%)	10(8)	15(12)	41(32)	50(38)
Potassium Average, mmol/L	4.04 (3.15～5.17)	4.15 (3.47～5.39)	4.11 (3.27～5.19)	4.28 (3.32～6.8)
Male, n(%)	90(71)	93(73)	91(71)	90(73)
Age, years	56(18～93)	59(18～93)	60(18～91)	57(18～101)
ICU hospitalization days	15(3～66)	19(3～99)	21(2～86)	16(2～67)
SOFA	7(2～16)	7(2～16)	8(2～17)	8(2～18)
Glucose SD	1.7(0～7.25)	1.7(0～5.72)	2.1(0～13.23)	2.2(0～5.87)
Glucose CV(%)	18(0～53)	18(0～73)	21(0～55)	22(0～64)
Glucose Average, mmol/L	9.1(3.5～17.3)	9.4(5.2～17.7)	9.4(5.1～24.2)	9.7(3～22.7)
Glucose maximum, mmol/L	11.4(0～26.5)	11.4(0～25.1)	12.1(0～41.6)	12.3(0～30.3)
Glucose minimum, mmol/L	6.7(0～17.1)	6.9(0～14.9)	6.2(0～13.9)	6.4(0～16.6)

Urine output，ml	1616(0～4950)	1626(0～4550)	1412(0～4500)	1322(0～5700)
Hemodialysis, n(%)	16(13)	23(18)	38(29)	47(36)
Insulin, u	13(0～108)	14(0～120)	16(0～124)	13(0～90)
eGFR	106（4.5～589）	110（5.2～563）	94（6～462）	74（2.5～317）
Potassium Chloride, g	1(0～11)	0.7(0～9)	1(0～10)	0.7(0～11)
Furosemide, mg	11(0～120)	14(0～120)	10(0～80)	10(0～50)
PH	7.42(6.9～7.6)	7.43(7.12～7.6)	7.41(7.17～7.63)	7.39(6.96～7.58)
Oxygenation Index, mmHg	233(38～598)	231(31～649)	227(33～497)	218(43～514)
Creatinine, μmol/L	132(16～1046)	132(17～999)	158(19～708)	200(27～1705)
Bilirubin, μmol/L	28(2～337)	23(2.3～206)	32(2～301)	42(3～378)
Platelet，10*9/L	225(3～620)	215(7.1～718)	180(8～1020)	203(2～3206)

Table 2 Comparison of potassium concentration groups

Potassium group (mmol/l)	**3.0-3.5**	**>3.5-4.0**	**>4.0-4.5**	**>4.5-5.0**	**>5.0-5.5**	**>5.5**	**Total**
Number of patients, n	21	165	229	79	10	2	506
Number of death，n	10	52	91	44	8	1	206
Mortality rate (%)	47.6	31.5	39.7	55.7	80	50	40.7

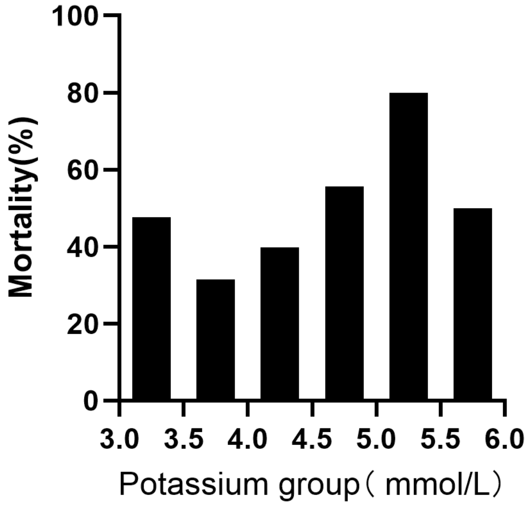

Figure 1 Mortality in different potassium concentration groups.

Table 3 Comparison of potassium concentration groups under different groups (Q1, Q2, Q3, Q4)

	**3.0-3.5**	**>3.5-4.0**	**>4.0-4.5**	**>4.5-5.0**	**>5.0-5.5**	**>5.5**
	Mortality rate in (%)					

Q 1 (CV≤9.17%）	16.7	17.8	23.1	20	100	0
Q 2 (9.17%＜CV≤11.43%）	100	20.8	44.7	63.6	75	0
Q 3 (11.43%＜CV≤14.37%）	42.9	48.9	45.8	52.9	100	0
Q 4 (CV＞14.37%）	66.7	50	48.3	63.3	50	50
	Total numbers，n					
Q 1 (CV≤9.17%）	6	45	65	10	1	0
Q 2 (9.17%＜CV≤11.43%）	2	53	47	22	4	0
Q 3 (11.43%＜CV≤14.37%）	7	43	59	17	3	0
Q 4 (CV＞14.37%）	6	24	58	30	2	2

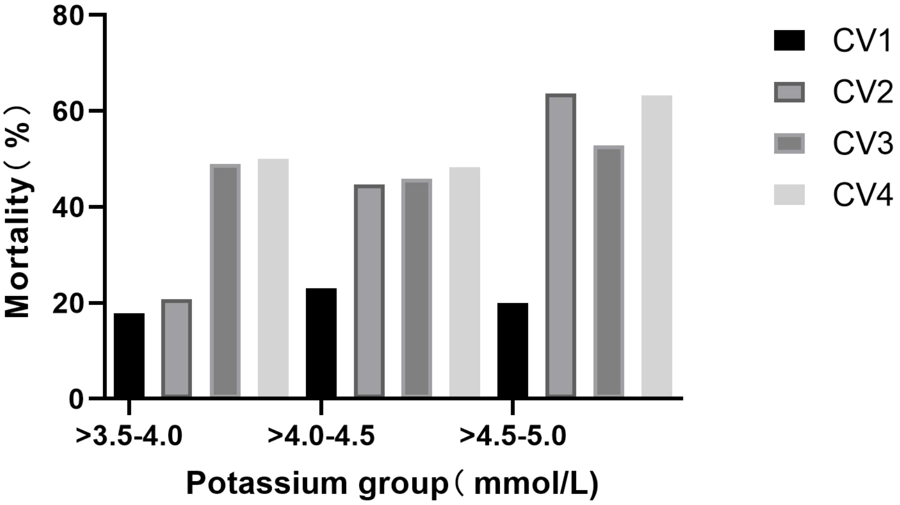

Figure 2 Chart comparing potassium concentration groups under potassium variability groups. We can see that mortality increases with potassium variability when potassium concentrations are in the range of 3.5-5mmol/L
