# Supplementary material for: The impact of serum potassium ion variability on 28-day mortality in ICU patients
Source: PLoS One. 2024 Nov 4;19(11):e0310046. doi: 10.1371/journal.pone.0310046 (PMC11534218; doi:10.1371/journal.pone.0310046)
Supplement: S4 Appendix — (PDF) [file pone.0310046.s004.pdf]

Appendix 4 Changes in potassium concentration or CV within 28 days

Table 1 Potassium concentration changes and CV changes every 12 hours

Hours	K(mmol/L)	CV(%)
12	3.99	
24	4.06	3.26
36	4.06	
48	4.05	4.64
60	4.07	
72	4.03	4.16
84	4.12	
96	4.14	4.35
108	4.13	
120	4.13	3.44
132	4.01	
144	4.10	4.25
156	4.12	
168	4.17	2.83
180	4.18	
192	4.16	3.63
204	4.19	
216	4.14	3.44
228	4.15	
240	4.17	3.40
252	4.22	
264	4.17	3.18
276	4.13	
288	4.23	5.18
300	4.17	
312	4.14	4.93
324	4.21	
336	4.16	4.96
348	4.20	
360	4.20	4.75
372	4.21	
384	4.26	5.18
396	4.24	
408	4.17	5.79

420	4.19	
432	4.21	4.96
444	4.20	
456	4.17	5.15
468	4.18	
480	4.16	5.35
492	4.17	
504	4.27	5.43
516	4.19	
528	4.19	5.00
540	4.21	
552	4.21	5.01
564	4.20	
576	4.29	4.20
588	4.23	
600	4.16	4.53
612	4.09	
624	4.21	6.25
636	4.10	
648	4.14	4.28
660	4.20	
672	4.12	5.38

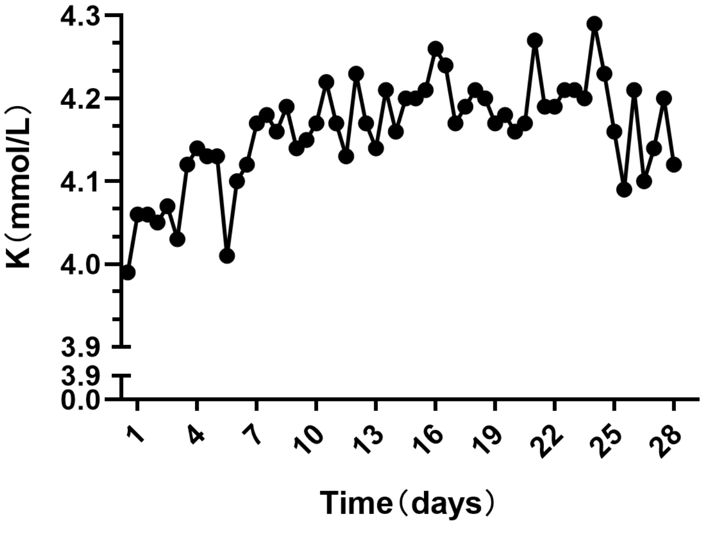

Fig1：28 day average potassium concentration and its trend in ICU hospitalization

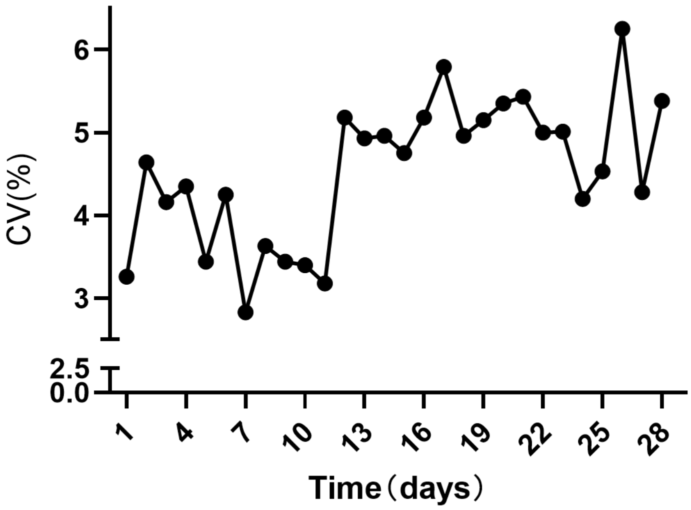

Fig2：The variability and trend of average potassium levels during 28 days of ICU stay

Table 2 Comparison between normal and abnormal potassium concentration groups

Days	Normal group CV(%)	Abnormal group CV(%)
1	3.58	4.88
2	4.36	6.08
3	3.37	5.09
4	3.26	5.50
5	2.53	4.61
6	3.34	5.17
7	2.07	3.11
8	3.07	3.89
9	2.85	3.60
10	2.83	3.95
11	2.64	3.93
12	4.70	6.62
13	4.91	5.86
14	4.73	6.14
15	4.46	5.60
16	5.26	6.46
17	6.08	7.04
18	5.14	5.88
19	5.27	6.63
20	5.25	6.23
21	4.88	6.36
22	4.07	5.85

23	4.83	5.27
24	3.64	5.22
25	4.72	5.26
26	5.63	6.91
27	3.98	4.27
28	4.65	5.97
Average value	3.88	5.41
Total number of people, n(%)	334（66）	172（34）
Mortality, n(%)	112（36.5）	84（48.8）

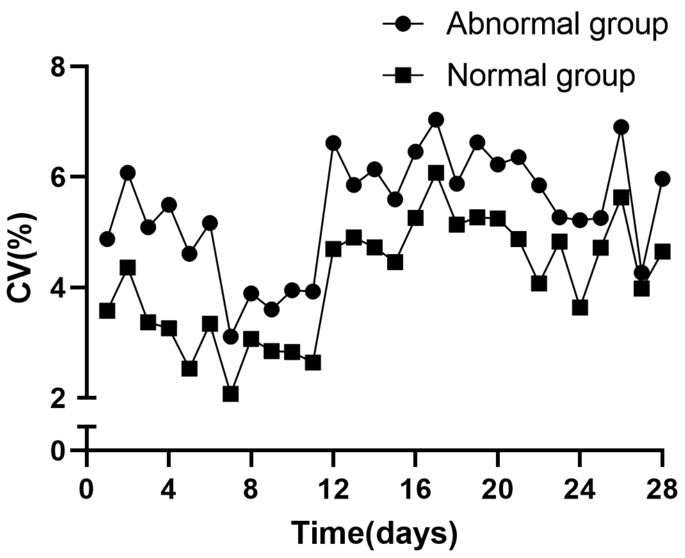

Figure 3 The potassium abnormality group consists of patients with a single potassium measurement value greater than 6.0 mmol/L or potassium<3.0 mmol/L within 28 days of ICU admission. The normal potassium group includes patients with a single potassium measurement of 3.0-6.0 mmol/L within 28 days of ICU admission.

Table 3 CV changed within 28 days

	**Average CV（%）**	
**Days**	**Survival**	**Death**
1	3.58	2.81
2	4.36	5.00
3	3.37	5.32
4	3.26	5.81
5	2.53	4.87
6	3.34	5.56
7	2.07	4.15
8	3.07	4.63

9	2.85	4.46
10	2.83	4.40
11	2.64	4.14
12	4.70	6.11
13	4.91	4.98
14	4.73	5.40
15	4.46	5.25
16	5.26	5.04
17	6.08	5.32
18	5.14	4.71
19	5.27	4.98
20	5.25	5.49
21	4.88	6.14
22	4.07	6.12
23	4.83	5.23
24	3.64	4.88
25	4.72	4.27
26	5.63	7.12
27	3.98	4.70
28	4.65	6.40
The overall average CV of the survival group	4.15	-
The total average CV of the death group	-	5.12

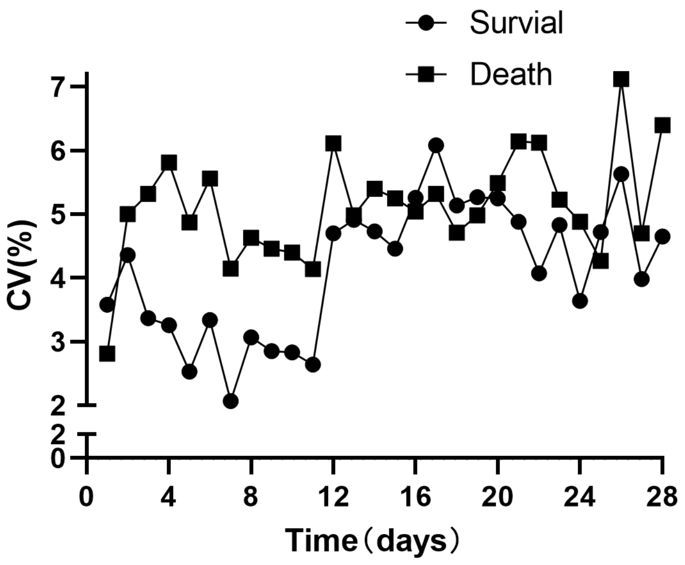

Fig4：Comparative Trends of Coefficient of Variation (CV) in the Death and Survival Groups Over 28 Days
