# Supplementary material for: The impact of serum potassium ion variability on 28-day mortality in ICU patients
Source: PLoS One. 2024 Nov 4;19(11):e0310046. doi: 10.1371/journal.pone.0310046 (PMC11534218; doi:10.1371/journal.pone.0310046)
Supplement: S5 Appendix — (PDF) [file pone.0310046.s005.pdf]

Appendix 5 General information and comparison of death and survival groups

Table 1 Comparison of Dead and Surviving Patients

	Death group<br>Average (range)	Survival group<br>Average (range)	P
Number Of Patients，n(%)	206（41）	300（59）	-
Total CV Of Potassium(%)	14(6～41)	11(2～35)	0.00
Total SD Of Potassium	0.58(0.2～2.8)	0.46(0.05～1.46)	0.00
Heart Rate，beats per minute	101(50～194)	99(46～171)	0.46
Systolic Pressure，mmHg	128(50～221)	129(69～208)	0.55
Diastolic Pressure，mmHg	73(36～139)	72(33～129)	0.40
Mean Arterial Pressure，mmHg	92(41～166)	91(45～151)	0.70
Potassium Minimum，mmol/L	3.27(2.1～4.6)	3.24(1.7～4.5)	0.65
Potassium Maximum，mmol/L	5.43(3.9～9.6)	5.07(3.2～9.1)	0.00
Hyperkalemia，n(%)	46(22)	28(9)	0.00
Hypokalemia，n(%)	47(23)	66(22)	0.91
Potassium Average，mmol/L	4.23(3.32～6.8)	4.08(3.15～6.18)	0.00
Male，n(%)	157(76)	206(69)	0.06
Age，years	61(18～101)	56(18～93)	0.00
ICU Hospitalization Days	16(2～86)	20(3～99)	0.00
SOFA	8(2～18)	7(2～17)	0.00
Glucose SD	1.92(0～6.42)	1.88(0～13.23)	0.38
Glucose CV(%)	20(0～57)	20(0～73)	0.97
Glucose Average，mmol/L	9.6(3～22.66)	9.19(4.85～24.19)	0.08
Glucose Maximum，mmol/L	12.19(0～27.8)	11.46(0～41.6)	0.06
Glucose Minimum，mmol/L	6.82(0～17.1)	6.33(0～15.6)	0.05
Urine Output，ml	1531(0～5700)	1467(0～4950)	0.79
Hemodialysis，n(%)	66(32)	55(18)	0.00
eGFR	83（2.5～442）	104（4.4～589）	0.00
Insulin，u	16(0～120)	13(0～124)	0.02
Potassium Chloride，g	0.7(0～11)	1(0～11)	0.26
Furosemide，mg	15(0～120)	9(0～120)	0.00
PH	7.41(6.85～7.6)	7.41(7.2～7.63)	0.98
Oxygenation Index, mmHg	228(31～649)	227(33～598)	0.87

Creatinine， μmol/L	192(19～1705)	132(16～1046)	0.00
Bilirubin， μmol/L	33(2～369)	30(2～378)	0.13
Platelet，10*9/L	197(7～3206)	211(2～763)	0.01
Coronary Heart Disease，n(%)	25(12)	29(10)	0.38
Cardiac Dysfunction，n(%)	20(10)	15(5)	0.04
Hypertension，n(%)	38(18)	88(29)	0.01
Cerebrovascular Diseases，n(%)	19(9)	11(4)	0.01
Chronic Obstructive Pulmonary Disease，n(%)	3(1)	5(2)	0.85
Diabetes，n(%)	18(9)	47(16)	0.02
Renal Insufficiency，n(%)	99(48)	119(40)	0.06
Shock，n(%)	83(40)	94(31)	0.04

Table 2 Comparison of General Information

	Average (range)
Total CV Of Potassium(%)	12(2～45)
Total SD Of Potassium	0.51(0.05～2.64)
Heart Rate, beats per minute	100(46～194)
Systolic Pressure, mmHg	129(50～221)
Diastolic Pressure, mmHg	73(33～139)
Mean Arterial Pressure, mmHg	91(41～166)
Potassium Minimum, mmol/L	3.24(1.7～4.6)
Potassium Maximum, mmol/L	5.22(3.2～9.6)
Hyperkalemia, n(%)	74(15)
Hypokalemia, n(%)	119(24)
Potassium Average, mmol/L	4.14(3.15～6.8)
Male, n(%)	363(72)
Age, years	58(18～101)
ICU Hospitalization Days	18(2～99)
SOFA	8(2～18)
Glucose SD	1.9(0～13.23)
Glucose CV(%)	20(0～73)
Glucose Average, mmol/L	9.36(3～24.19)
Glucose Maximum， mmol/L	11.76(0～41.6)
Glucose Minimum, mmol/L	6.53(0～17.1)
Urine output， ml	1493(0～5700)
Hemodialysis, n(%)	121(24)
eGFR	96（2.5～589）
Insulin, u	14.35(0～124)
Furosemide, mg	9.1(0～110)

Potassium Chloride, g	1.2(0～12)
PH	7.41(6.85～7.63)
Oxygenation Index, mmHg	228(31～649)
Creatinine, μmol/L	155(16～1705)
Bilirubin, μmol/L	31(2.1～378)
Platelet, 10*9/L	205(2～3206)
Coronary Heart Disease，n(%)	54(11)
Cardiac Dysfunction，n(%)	35(7)
Hypertension，n(%)	126(25)
Cerebrovascular Diseases，n(%)	30(6)
Chronic Obstructive Pulmonary Disease，n(%)	8(2)
Diabetes，n(%)	65(13)
Renal Insufficiency，n(%)	218(43)
Shock，n(%)	177(35)
