# Supplementary material for: The impact of serum potassium ion variability on 28-day mortality in ICU patients
Source: PLoS One. 2024 Nov 4;19(11):e0310046. doi: 10.1371/journal.pone.0310046 (PMC11534218; doi:10.1371/journal.pone.0310046)
Supplement: S6 Appendix — (PDF) [file pone.0310046.s006.pdf]

Appendix 6 Binary logistic regression model and normality test and single factor analysis

Table1: Association of potassium ion variability group and potassium group with in-hospital mortality (Covariates: Age, Male, ICU hospitalization days)

	Death, n(%)	Model1		Model2	
		OR (95%CI)	P-VALUE FOR TRENT	OR (95%CI)	P-VALUE FOR TRENT
Q1 (CV≤9.17%)	27(21)	1.00 (Reference)	—	1.00 (Reference)	—
Q2 (9.17%＜CV≤11.43%)	51(40)	2.85 (1.59～5.09)	P＜0.01	3.01 (1.66～5.45)	P＜0.01
Q3 (11.43%＜CV≤14.37%)	63(49)	4.39 (2.49～7.75)	P＜0.01	4.87 (2.7～8.77)	P＜0.01
Q4 (CV＞14.37%)	70(54)	5.12 (2.9～9.05)	P＜0.01	5.4 (3.02～9.66)	P＜0.01
Covariates					
Male				1.53(1～2.34)	P＜0.05
Age				1.02 (1.01～1.03)	P＜0.01
ICU hospitalization days				0.98 (0.96～0.99)	P＜0.01
	Death, n(%)	Model3		Model4	
		OR (95%CI)	P-VALUE FOR TRENT	OR (95%CI)	P-VALUE FOR TRENT
3.5-4.0mmol/L	52(32)	1.00 (Reference)	—	1.00 (Reference)	—
4.0-4.5mmol/L	91(40)	1.3 (0.86～1.97)	P=0.22	1.28 (0.84～1.96)	P=0.26
4.5-5.0Mmol/L	44(56)	2.5 (1.44～4.33)	P＜0.01	2.27 (1.3～3.97)	P＜0.01
Covariates					

Male	1.64（0.89 ~ 3.02)	P=0.18
Age	1.02（1.01 ~ 1.04)	P=0.01
ICU hospitalization days	0.99（0.97 ~ 1.01)	P < 0.01

	Model5		Model6	
	OR (95%CI)	P-VALUE FOR TRENT	OR (95%CI)	P-VALUE FOR TRENT
Q1	1.00 （Reference）	—	1.00 （Reference）	—
Q2	3.37(1.61 ~ 7.05)	P < 0.01	2.06(1.01 ~ 4.2)	P < 0.01
Q3	6.96(3.03 ~ 16.01)	P < 0.01	3.24(1.43 ~ 7.36)	P < 0.01
Q4	6.54(2.2 ~ 19.43)	P < 0.01	2.81(1 ~ 7.89)	P < 0.01
Covariates	OR (95%CI)	P-VALUE FOR TRENT	OR (95%CI)	P-VALUE FOR TRENT
Potassium Minimum Value	0.94(0.29 ~ 3.02)	P=0.91	0.73(0.32 ~ 1.65)	P=0.45
Potassium Maximum Value	0.62(0.31 ~ 1.22)	P=0.17	0.53(0.29 ~ 0.97)	P=0.04
Hyperkalemia	3.35(1.28 ~ 8.8)	P < 0.01	4.98(2.34 ~ 10.61)	P < 0.01
Hypokalemia	0.86(0.4 ~ 1.86)	P=0.71	0.68(0.34 ~ 1.36)	P=0.27
Potassium Average Value	2.19(0.66 ~ 7.29)	P=0.2	2.99(1.05 ~ 8.48)	P=0.04
Male	1.15(0.69 ~ 1.92)	P=0.59	1.11(0.67 ~ 1.82)	P=0.69
Age	1.02(1.01 ~ 1.04)	P < 0.01	1.03(1.01 ~ 1.04)	P < 0.01
ICU hospitalization days	0.98(0.96 ~ 1)	P < 0.01	0.98(0.96 ~ 1)	P < 0.01
SOFA	1.05(0.96 ~ 1.14)	P=0.27	1.07(0.98 ~ 1.17)	P=0.13
Glucose SD	1.18(0.37 ~ 3.72)	P=0.78	1.48(0.48 ~ 4.6)	P=0.5
Glucose CV	1.4(0 ~ 2008.21)	P=0.93	1.36(0 ~ 1480.92)	P=0.93
Glucose Average	1.15(0.81 ~ 1.64)	P=0.42	1.2(0.85 ~ 1.7)	P=0.31
Glucose maximum	0.84(0.61 ~ 1.14)	P=0.25	0.76(0.56 ~ 1.04)	P=0.08
Glucose minimum	1.11(0.79 ~ 1.58)	P=0.54	1.14(0.8 ~ 1.64)	P=0.46
Urine output	1(1 ~ 1)	P < 0.01	1.01(1 ~ 1.07)	P < 0.01
Hemodialysis	2.27(1.22 ~ 4.22)	P < 0.01	2.34(1.25 ~ 4.37)	P < 0.01
eGFR	1(1 ~ 1.01)	P=0.02	1(1 ~ 1.01)	P=0.03
Insulin	1(0.99 ~ 1.01)	P=0.56	1(0.99 ~ 1.01)	P=0.58
Potassium chloride	0.99(0.97 ~ 1)	P=0.03	0.99(0.98 ~ 1)	P=0.04
Furosemide	1.02(1.01 ~ 1.04)	P < 0.01	1.02(1.01 ~ 1.04)	P < 0.01
PH	4.47(0.28 ~ 71.36)	P=0.29	2.81(0.17 ~ 46.94)	P=0.47

Model 1: univariable model.
Model 2: multivariable model adjusted for age, male, ICU hospitalization days
Model 3: univariable model.
Model 4: multivariable model adjusted for age, male, ICU hospitalization days
Model 5: Model after adjusting for confounding factors(Potassium Minimum Value、Potassium Maximum Value、Hyperkalemia、Hypokalemia、Potassium Average Value、male、Age、ICU hospitalization days、SOFA、Glucose SD、Glucose CV、Glucose Average、Glucose maximum、Glucose minimum、Urine output、Hemodialysis、eGFR、Insulin、potassium chloride、Furosemide、PH).
Model 6: Model after multiple imputation and adjusting for confounding factors (Potassium Minimum Value、Potassium Maximum Value、Hyperkalemia、Hypokalemia、Potassium Average Value、male、Age、ICU hospitalization days、SOFA、Glucose SD、Glucose CV、Glucose Average、Glucose maximum、Glucose minimum、Urine output、Hemodialysis、eGFR、Insulin、potassium chloride、Furosemide、PH).

Table 2: Statistical Models Excluding Cases of potassium abnormality（K＞6mmol/L or K＜3mmol/L) during ICU period

Quartile	Model7		Model8		Model9	
	OR (95%CI)	P-VALUE FOR TRENT	OR (95%CI)	P-VALUE FOR TRENT	OR (95%CI)	P-VALUE FOR TRENT
Q1 (CV≤9.17%)	1.00（Reference）	—	1.00（Reference）	—	1.00（Reference）	—
Q2 (9.17% < CV ≤11.43%)	1.49（1.49～4.96）	P < 0.01	1.62（1.62～5.54）	P < 0.01	1.44（1.44～5.01）	P < 0.01
Q3 (11.43% < CV ≤14.37%)	2.62（2.62～8.63）	P < 0.01	2.49（2.49～9.04）	P < 0.01	2.34（2.34～8.77）	P < 0.01
Q4 (CV > 14.37%)	1.98（1.98～7.43）	P < 0.01	2.57（2.57～9.37）	P < 0.01	1.33（1.33～6.84）	P=0.01
Male	1.04（1.04～2.65）	P=0.03	0.97（0.97～2.68）	P=0.07	0.96（0.96～2.94）	P=0.07
Age	1.01（1.01～1.03）	P < 0.01	1（1～1.03）	P=0.03	1（1～1.03）	P=0.02

ICU hospitalization days	0. 97 (0. 97～1)	P < 0.01	0. 96 (0. 96 ～1)	P < 0.01	0. 96 (0. 96 ～1)	P < 0.05

Model 7: A model that excludes Cases of potassium abnormality（K > 6mmol/L) during ICU period and adjusts for confounding factors (age, male, ICU hospitalization days).

Model 8: A model that excludes Cases of potassium abnormality（K < 3mmol/L) during ICU period and adjusts for confounding factors (age, male, ICU hospitalization days).

Model 9: A model that excludes Cases of potassium abnormality（K > 6mmol/L and K < 3mmol/L) during ICU period and adjusts for confounding factors (age, male, ICU hospitalization days).

Table 3 Excluding check-in to ICU<4

Quartile	Model10		Model11	
	OR (95%CI)	P-VALUE FOR TRENT	OR (95%CI)	P-VALUE FOR TRENT
Q1 (CV≤9.17%)	1.00 (Reference)	—	1.00 (Reference)	—
Q2 (9.17% < CV≤11.43%)	3. 28 (1. 71～6. 31)	P < 0.01	3. 25 (1. 67～6. 33)	P < 0.01
Q3 (11.43% < CV≤14.37%)	5. 34 (2. 81～10. 17)	P < 0.01	5. 63 (2. 9～10. 93)	P < 0.01
Q4 (CV > 14.37%)	6. 47 (3. 39～12. 36)	P < 0.01	6. 61 (3. 41～12. 8)	P < 0.01
Male			1. 33 (0. 84～2. 11)	P=0.22
Age			1. 02 (1. 01～1. 04)	P < 0.01
ICU hospitalization days			0. 98 (0. 97～1)	P=0.02

Model 10: Excluding unadjusted models for patients who stayed in the ICU for less than 4 days.

Model 11: A model that excluded ICU stay<4 days and adjusted for confounding factors (age, male, ICU hospitalization days)

Table 4 Statistical model excluding patients with hemodialysis

Quartile	Model12		Model13	
	OR (95%CI)	P-VALUE	OR (95%CI)	P-VALUE

		FOR TRENT		FOR TRENT
Q1 (CV≤9.17%)	1.00 （Reference）	—	1.00 （Reference）	—
Q2 (9.17%＜CV≤11.43%)	2.78(1.45～5.34)	P＜0.01	2.87(1.47～5.59)	P＜0.01
Q3 (11.43%＜CV≤14.37%)	4.41(2.31～8.41)	P＜0.01	4.84(2.48～9.44)	P＜0.01
Q4 (CV＞14.37%)	5.12(2.64～9.95)	P＜0.01	5.25(2.67～10.34)	P＜0.01
Male			1.48(0.91～2.41)	P=0.11
Age			1.01(1～1.03)	P＜0.05
ICU hospitalization days			0.98(0.96～1)	P＜0.05

Model 12: univariable model.

Model 13: multivariable model adjusted for age, male, ICU hospitalization days

Table 5 : Normality test and single factor analysis table

Average potassium value (mmol/L)	Normality test	variable	Normality test	Univariate analysis
12h	0.84	Total CV	0.00	0.00
24h	0.25	Total SD	0.00	0.00
36h	0.00	Potassium concentration grouping	0.00	0.00
48h	0.00	heart rate	0.00	0.46
60h	0.39	systolic pressure	0.00	0.55
72h	0.20	diastolic pressure	0.00	0.40
84h	0.77	mean arterial pressure	0.00	0.70
96h	0.42	Potassium minimum value	0.00	0.52
108h	0.39	Potassium maximum value	0.00	0.00
120h	0.44	Average potassium value	0.00	0.00
132h	0.03	Male	0.00	0.83
144h	0.05	Age	0.00	0.00
156h	0.71	ICU hospitalization days	0.00	0.06
168h	0.01	SOFA	0.00	0.00
180h	0.38	Glucose SD	0.00	0.00
192h	0.57	Glucose CV	0.00	0.00
204h	0.45	Glucose Average	0.00	0.38
216h	0.77	Glucose Maximum	0.00	0.97
228h	0.76	Glucose Minimum	0.00	0.08
240h	0.07	Urine output	0.00	0.06
252h	0.03	Hemodialysis	0.00	0.05
264h	0.47	eGFR	0.00	0.52
276h	0.31	insulin	0.00	0.79
288h	0.06	potassium chloride	0.00	0.00
300h	0.09	Furosemide	0.00	0.02
312h	0.05	PH	0.00	0.26
324h	0.10	Oxygenation Index	0.00	0.00
336h	0.03	Creatinine	0.00	0.99
348h	0.60	Bilirubin	0.00	0.87
360h	0.00	Platelet	0.00	0.00
372h	0.01	Coronary Heart Disease	0.00	0.13
384h	0.00	Cardiac Dysfunction	0.00	0.02
396h	0.00	Hypertension	0.00	0.38
408h	0.39	Cerebrovascular Diseases	0.00	0.04
420h	0.22	Chronic Obstructive Pulmonary Disease	0.00	0.01
432h	0.18	Diabetes	0.00	0.01

444h	0.02	Renal Insufficiency	0.00	0.85
456h	0.98	shock	0.00	0.02
468h	0.58			
480h	0.72			
492h	0.23			
504h	0.01			
516h	0.46			
528h	0.66			
540h	0.76			
552h	0.49			
564h	0.07			
576h	0.02			
588h	0.85			
600h	0.95			
612h	0.35			
624h	0.15			
636h	0.02			
648h	0.90			
660h	0.72			
672h	0.16			
