# Supplementary material for: The impact of serum potassium ion variability on 28-day mortality in ICU patients
Source: PLoS One. 2024 Nov 4;19(11):e0310046. doi: 10.1371/journal.pone.0310046 (PMC11534218; doi:10.1371/journal.pone.0310046)
Supplement: Attachment — Submitted filename: Response to Reviewers.pdf [file pone.0310046.s007.pdf]

Dear Dr Tatsuo Shimosawa:

On behalf of my co-authors, we thank you very much for giving us an opportunity to revise our manuscript, we appreciate editor and reviewers very much for their positive and constructive comments and suggestions on our manuscript entitled “The Impact of Serum Potassium Ion Variability on 28-Day Mortality in ICU Patients”.

We have carefully studied the opinions of the reviewers and The paper has been revised. We have tried our best to revise our manuscript according to the comments. Attached please find the revised version, which we would like to submit for your kind consideration.

We would like to express our great appreciation to you and reviewers for comments on our paper. Looking forward to hearing from you.

Thank you and best regards.

Yours sincerely,

YuChou Zhang

We thank the reviewers for their valuable comments, which certainly help us to improve the quality of our manuscript. All comments are addressed on a point-by-point basis below, where letters C & R denote Comment and Response, respectively. The modifications of the text, figures and tables have been highlighted.

Reviewer#1:

C:1)Since it is well known that the portality is associated with extreme values of sK,in order to exclude the effect of evere hypo-ofhepercalemia and termsofortality, I would recommend checking the correlation between CV and moratality only in those without experiencing extreme values(for example,sK<3,>6)inaddition to wholel analysis.This analysis should serve as a sensitivy analysis.

R:1)Thank you for your feedback, I agree with your point of view. Therefore, I established three statistical models. Patients with K>6, K<3, K>6, and K<3 were excluded and reanalyzed. Table 2 in Appendix 6 shows that all three statistical models are significant.

C:2)Significant change in sK can occur especially in those with hemodialysis or CRRT. I would recommend to add renal replacement therapy (yes or no) as a variable in the analysis.

R:2)Thank you for your suggestion. I agree with your viewpoint. We added the variable of hemodialysis to the statistical model and found P<0.01 (model 4 in Appendix 6). Moreover, after excluding patients undergoing hemodialysis and reanalyzing, it was found to still be significant (Table 4 in Appendix 6).

C:3)Also, sK change can be exaggerated in those with lreduced kidney function. It looks like "renal insufficiency" is rare in the study population, how you define "renal insufficiency"? I would like to see the effect of degree of eGFR to the CV of sK and also to the mortality.

R:3)Thank you for your valuable feedback. We have made improvements based on your feedback. ICD-10-diagnoses were determined by physicians depending on the guidelines valid at the time. ICD-10-GM derived covariates are as followed: Renal Insufficiency N18.001-N18.004. eGFR was calculated based on age, gender, and creatinine levels. After incorporating the covariate eGFR into the statistical model, it showed significant differences (P=0.03) (Model 5). In addition,

Appendix 5 shows a significant difference in eGFR between the death group and the inventory group.

Reviewer#2

C:1） Overall, the correlation between K values and mortality has been reported in large clinical studies.The total number of patients is 237, which is too small to statistically correlate. It is necessary to increase the number of patients to examine the correlation.

R:1） Thank you for your suggestion. We agree with your opinion, so we have increased the sample size and reanalyzed the article.

C:2）The period of observation is slightly different and more detailed data is needed to correlate the one-week variation in CV of K-values with the risk of death during 28 days. (If possible, K values and CV values should also be listed for up to 28 days.)

R:2）Thank you for your suggestion. We agree with your viewpoint. We increased the observation period of K to 28 days, and then collected relevant data and reanalyzed it.

C:3） The K-adjustment method for dialysis patients is different from that for other patients, so separate analyses are recommended.

R:3） We agree with your viewpoint. Therefore, we excluded patients who had undergone blood purification from the statistical model and conducted reanalysis, and the results still showed significance (Table 4 in Appendix 6)

C:4） In Table 2 Covariates, P value is described from Male to PH, but OR (95%) is described. Confirmation is needed.

R:4） Thank you for your feedback. Table 2 describes OR (95% CI).

C:5） It is necessary to list the mean K values before admission to ICU and at 28 days.

R:5） Thank you for your suggestion. We collected potassium values within 28 days of ICU and reanalyzed them.

C:6）The report concludes that there is a significant difference by sex (male), but it is necessary to explain the reason for this in the discussion.

R:6） We agree with your opinion and have made revisions in the article. For the significance of men in the statistical model, relevant literature points out that male or female exposure may affect the incidence rate and prognosis of various critical diseases throughout the life cycle. Biological differences, sex hormone and cytokine responses may lead to observed gender differences, but the mechanisms underlying these gender differences cannot currently be explained. 根据诊断相关的性别平衡，ICU 患者死亡率的性别差异 - PMC (nih.gov)
